# Central vein stenosis in hemodialysis vascular access: clinical manifestations and contemporary management strategies

**DOI:** 10.3389/fneph.2023.1280666

**Published:** 2023-11-09

**Authors:** Gift Echefu, Ifeoluwa Stowe, Abdulkareem Lukan, Gaurav Sharma, Indranill Basu-Ray, London Guidry, Jon Schellack, Damodar Kumbala

**Affiliations:** ^1^ Division of Cardiovascular Medicine, The University of Tennessee Health Science Center, Memphis, TN, United States; ^2^ Department of Internal Medicine, Baton Rouge General Medical Center, Baton Rouge, LA, United States; ^3^ Department of Internal Medicine, Advocate Illinois Masonic Medical Center, Chicago, IL, United States; ^4^ Department of Nephrology, AIIMS Rishikesh, Rishikesh, India; ^5^ Department of Cardiology, AIIMS Rishikesh, Rishikesh, India; ^6^ Department of Cardiovascular Disease, Memphis Veterans Affairs Medical Center, Memphis, TN, United States; ^7^ Vascular Clinic of Baton Rouge, Baton Rouge, LA, United States; ^8^ Renal Associates of Baton Rouge, Baton Rouge, LA, United States

**Keywords:** central vein stenosis and obstruction, hemodialysis vascular access dysfunction, percutaneous angioplasty (PTA), indwelling catheter complications, endoluminal obstruction, Hemodialysis Reliable Outflow (HeRO) graft

## Abstract

Central venous stenosis is a significant and frequently encountered problem in managing hemodialysis (HD) patients. Venous hypertension, often accompanied by severe symptoms, undermines the integrity of the hemodialysis access circuit. In central venous stenosis, dialysis through an arteriovenous fistula is usually inefficient, with high recirculation rates and prolonged bleeding after dialysis. Central vein stenosis is a known complication of indwelling intravascular and cardiac devices, such as peripherally inserted central catheters, long-term cuffed hemodialysis catheters, and pacemaker wires. Hence, preventing this challenging condition requires minimization of central venous catheter use. Endovascular interventions are the primary approach for treating central vein stenosis. Percutaneous angioplasty and stent placement may reestablish vascular function in cases of elastic and recurrent lesions. Currently, there is no consensus on the optimal treatment, as existing management approaches have a wide range of patency rates.

## Introduction

Hemodialysis patients with end-stage renal disease need optimal vascular access to promote survival. The type of hemodialysis access and its maintenance significantly impact their mortality and quality of life. Functioning access is essential for the provision of appropriate hemodialysis (HD). In cases where an arteriovenous fistula cannot be placed, an arteriovenous graft is an alternative access. The tunneled, cuffed HD catheter is the least favored. Cuffed HD catheters are usually placed for the initiation of HD in patients with immature AV access or as a last resort in patients with no other vascular access alternatives (1–3). According to the Kidney Disease Outcome Quality Initiative (KDOQI), a CVC is acceptable for dialysis in the short term if an AV access has been created but is not ready for use, in patients with acute transplant rejection, or other complications requiring dialysis, peritoneal dialysis patients with complications that require short-term HD due to time-limited peritoneal rest or AV access complications that result in temporary non-use and patient scheduled for a living donor transplant in <90 days. Furthermore, it is acceptable for long-term use if the patient has had multiple failed prior AV accesses with no available options, limited life expectancy, or valid patient preference (4).

Although central venous stenosis and occlusion are common, they are often underdiagnosed, resulting in significant long-term effects, such as venous hypertension leading to inadequate dialysis delivery due to recirculation, reduced AVF maturation, and lower long-term patency rates, and superior vena cava syndrome. CVS increases with stiff non-cuffed catheters primarily if used for an extended period (1, 5, 6). Therefore, KDOQI guidelines recommend their use for periods not exceeding seven days (7). Nevertheless, central venous stenosis has been observed in patients with neither a central vein catheter nor a history of thrombogenic procedures (8). Once this condition develops, management becomes a challenge. Despite percutaneous intravascular intervention being regarded as the initial therapy of choice, the best management approach to achieve the highest patency still needs to be determined. This review aims to present current information regarding the pathophysiological mechanisms and risk factors that contribute to the development of central venous stenosis/occlusion. Additionally, the management strategies and the evidence regarding patency rates are discussed.

## Anatomy

The present review focuses primarily on obstruction of the thoracic central venous system, including the intrathoracic segments of the subclavian, brachiocephalic, internal jugular veins, and the superior vena cava. The standard definition for thoracic central veins refers to those located inferior to the thoracic outlet, central to the inner margin of the first rib, and superior to the diaphragmatic hiatus (9). Central veins are larger, with fewer valves and high flow rates compared to peripheral veins. They have unidirectional blood flow routes, but collaterals may develop in diseased states. Understanding the path of the central veins and their course through surrounding structures is crucial to comprehending why CVS occurs at these sites. The brachial and basilic veins unite at the inferior border of the teres major muscle to form the axillary vein. It then courses anterior to the subscapularis muscle, posterior to the pectoralis minor, and projects to the lateral border of the first rib, continuing as the subclavian vein. The subclavian vein enters the thoracic inlet just posterior to the clavicle, anterior to the first rib and costoclavicular space, where it unites with the internal jugular vein (from the head and neck) to become the brachiocephalic vein (BCV). The BCV on both sides then merges to form the superior vena cava (10). Central venous catheters may terminate in the superior vena cava (SVC), inferior vena cava (IVC), or right atrium. This review does not address several morphological variants of the central thoracic veins rarely associated with reduced function or CVS.

**Figure 1:**
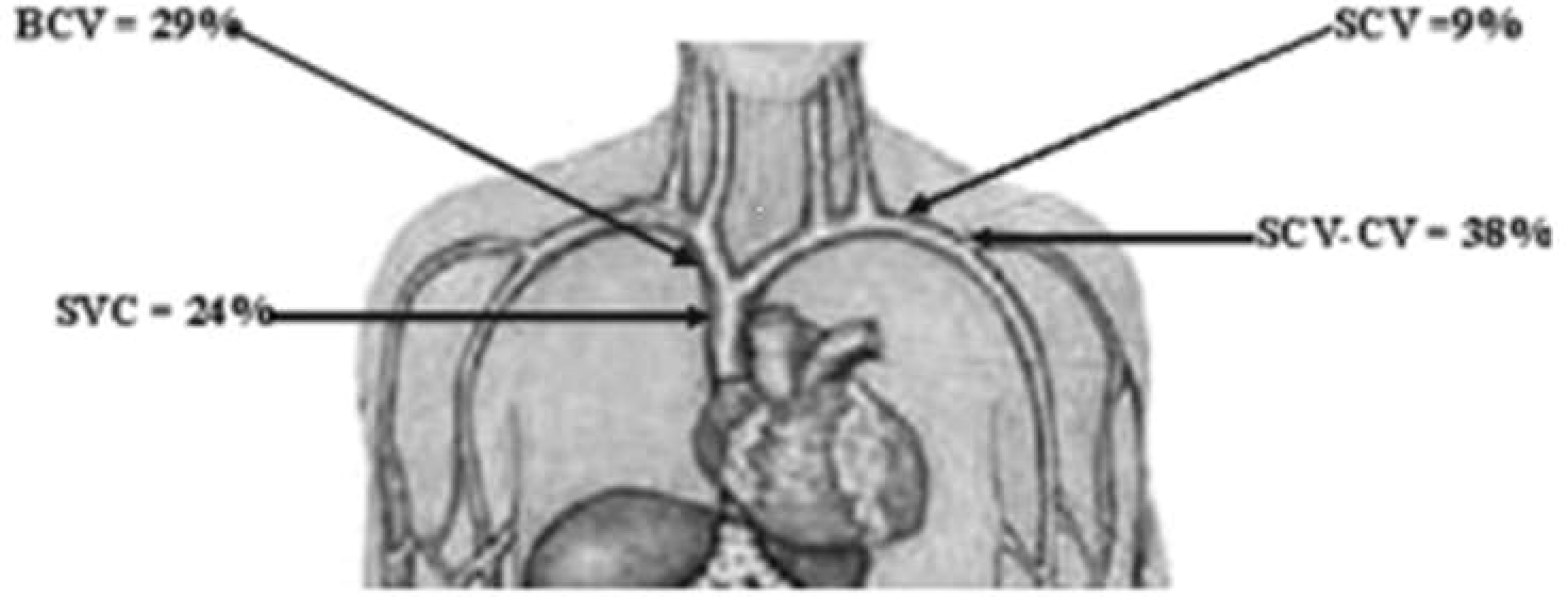
Anatomic distribution of CVS. CVS, central vein stenosis; SCV, subclavian vein; BCV, brachiocephalic vein; SVC, superior vena cava; SCV-CV, SCV, and subclavian-cephalic vein junction. Image is derived from MacRae et al. (11).

McCrae et al. detailed the anatomic distribution of CVS based on the study of 133 HD patients with venogram-confirmed CVS (11). According to the authors, most CVS lesions are located at the junction of the subclavian and cephalic veins (38%), followed by the brachiocephalic vein (29%), and then the superior vena cava (24%). The free segment of the subclavian vein is the least involved (**Figure 1**) (11).

## Epidemiology

In 2019, over 130,000 individuals in the United States were newly diagnosed with end-stage renal disease (ESRD) with an adjusted incidence of 386 parts per million, and 85% of these individuals initiated in-center hemodialysis (HD) (12). The prevalence of CVS varies, ranging from 4.3-41% depending on the population studied. The true prevalence may be underestimated since patients without suggestive symptoms are not routinely screened (1, 11, 13–15).

In a study to determine the prevalence of CVS among HD patients who underwent venography for access-related complaints, 41% of these patients exhibited significant CVS on venogram in contrast, when the study population consisted of stage 4 &5 pre-dialysis chronic kidney disease (CKD) and ESRD patients on HD who underwent venographic vein mapping, the prevalence of central vein stenosis was 10% for the whole group, 13% among patients with tunneled central venous dialysis catheters and 2% among the pre-dialysis patients (11, 14).

A retrospective study of ESRD patients on HD reported CVS in 4.3% (120) of 2811 patients at a median dialysis vintage of 2.9 years (1). Further study analysis to identify the rates of CVC-associated CVS revealed that in a subset review of 500 patients with such history, CVS was noted in about 34 (6.8%), at the rate of 2.2 per 100 patient-years. In addition, the frequency of central venous stenosis increased with the number of previous catheters (RR, 2.2; 95% CI, 1.6 to 2.9), pacemaker implantation RR 3.9; 95% CI, 1.7 to 8.9) and decreased with age (RR, 0.7 per decade; 95% CI, 0.6 to 0.8) (1).

## Risk factors

Factors that have been independently associated with CVS include the use of tunneled hemodialysis catheters, duration of CVC dependence, the number of CVC placements, presence of cardiac devices, younger age at dialysis initiation, previous history of fistula or graft, and history of prior kidney transplant (11, 14–16). Increased risk for the development of CVS is directly related to the placement of intravascular catheters and devices. Insertion of CVC at the time of HD initiation is a common practice. Based on the report from the United States Renal Data System (USRDS), the percentage of patients initiating HD with a catheter (with or without a maturing graft or fistula) in 2019 was 81.8%, while the percentage of patients initiating HD with a catheter alone was 67.8% (12). The incidence of CVS associated with HD catheters varies relative to the type of catheter, duration of use, and location of the vessel accessed (16). The lifetime number of CVC placements is independently associated with a high risk of CVS (11, 16). A single-center study of 106 HD patients found a prevalence of 28.3% (17) of CVS cases.

The prevalence of CVS was 3.4%, 29.4%, and 53.8% among patients with a history of 0-1 vs. 2-3 vs 4 or more central venous catheter placements, respectively. Furthermore, CVS was more prevalent in patients with one prior or current subclavian vein catheterization than in patients without catheter placement in this vein (respectively 47.8% vs. 22.0%, p = 0.02). No similar trend was observed in patients with previous or current jugular or femoral venous catheterizations (16). The right internal jugular vein is the preferred site for hemodialysis catheter placement due to the relatively lower incidence of CVS associated with this site. This is attributable to the straight course of the catheter as it goes through the right internal jugular vein into the right brachiocephalic vein and then the superior vena cava (18–21). Despite the lower incidence of CVS associated with IJ catheters, they are associated with CVS as high as 25-40%, according to study reports (18–21). The subclavian vein has the highest incidence of CVS compared to other vessels due to the mechanical effect of the catheter relative to vascular anatomy (16, 22). Catheter placement for a longer duration increases the risk for CVS. Therefore, shorter terms are usually recommended (11, 22). A prospective study investigating the impact of short-term hemodialysis catheter placement on central veins reported a 14% (in 8 patients) incidence of CVS at a mean dwell time of 21 days (21). MacRae et al. also noted dialysis vintage and previous HD catheter use as associated risks for CVS (11).

Peripherally inserted central catheters are associated with a high incidence of venous stenosis in the peripheral (cephalic thrombus) and central veins (23). In a review analysis of angiographic studies performed pre- and post-PICC placement in 150 patients, 4.8% had central vein stenosis, and 2.7% had central venous occlusion (24). Considering the high incidence of thrombosis associated with PICC placement in established or prospective patients for HD, an alternative means of access should be explored to preserve vascular longevity. Furthermore, there may be merits in pursuing upper extremity venography before placing permanent HD access in patients with a history of PICC line placement. Similarly, placement of cardiac implantable devices (such as cardiac pacemakers and implantable cardioverter defibrillator devices) through the transvenous approach is associated with high rates of (25-64%) CVS in the general population (23–27).

## Pathomechanisms of central venous stenosis

**Figure 2:**
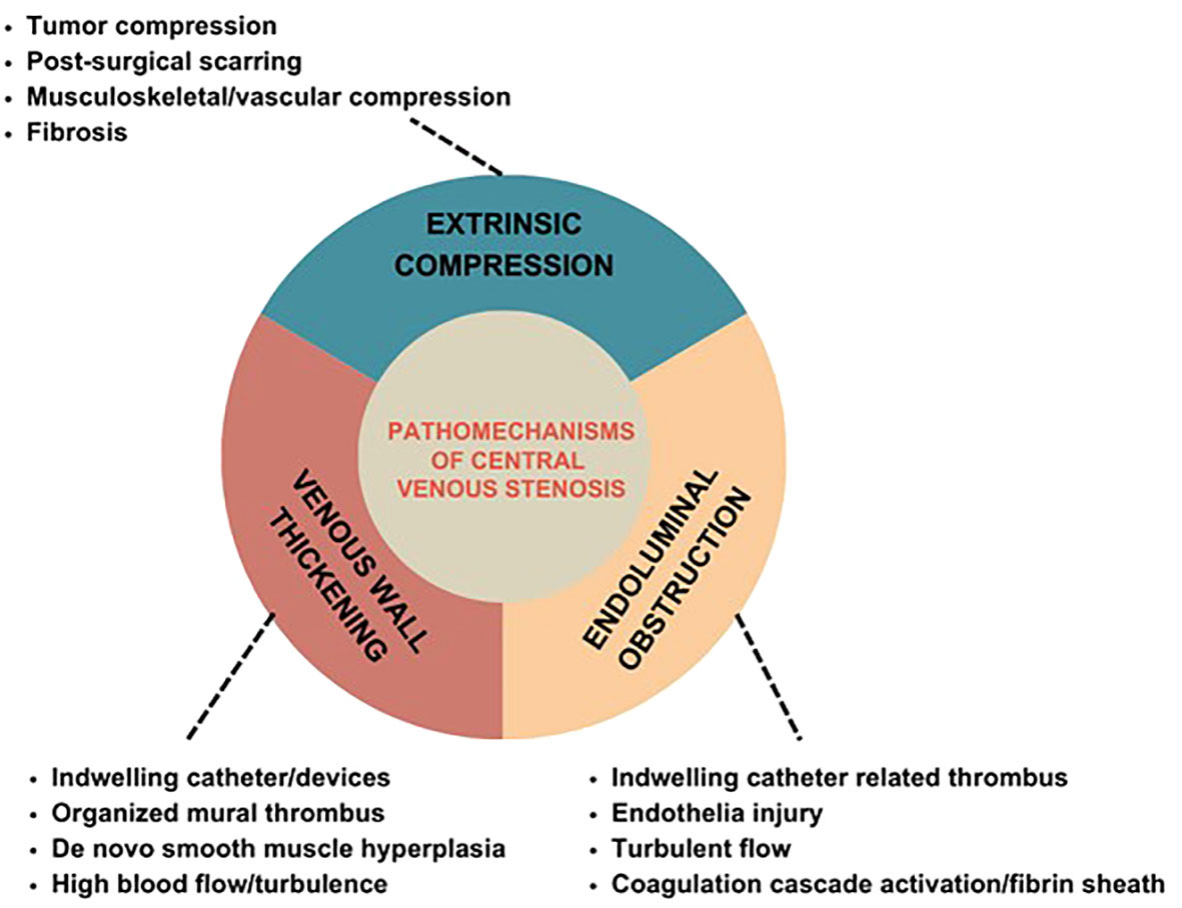
Pathomechanisms of Central Venous Stenosis.

CV stenosis could develop from thoracic inlet syndrome, extrinsic compression, and previous clavicle or pacemaker wire fracture. Among patients with a history of CVC, it is thought to be a consequence of endothelial injury with changes in the vessel wall that result in microthrombi, smooth muscle proliferation, and, ultimately, stenosis.3-5 Thus, the three primary mechanisms contributing to central venous obstruction include venous wall thickening, endoluminal obstruction, and extrinsic compression (**Figure 2**). It is common for these mechanisms to overlap to result in clinically significant CV occlusions.

### Venous wall thickening

Wall thickening is the most common mechanism for CVS. It could result from injury due to indwelling venous catheters or devices, organized mural thrombus or fibrosis, and *de novo* smooth muscle hyperplasia with no antecedent injury (28). Tunneled hemodialysis catheter and the presence of cardiac rhythm devices are independently associated with CVS. The pathogenesis of venous wall disease in vessels with CVC is a multi-step process. Firstly, there is consequent damage to the vessel at the CVC insertion site. This, combined with micro-injuries to the endothelium resulting from movement of the indwelling catheter, induces an inflammatory response and activation of the coagulation cascade leading to platelet activation and aggregation (17, 29–31). In a swine model, researchers identified cells in the venous neointima that were positive for alpha-smooth muscle actin, CD68, Ki67, smoothelin, and vimentin (32). Clots may develop along the thrombogenic catheter and form a sheath-like encasement to which fibrin attaches, the infiltration of smooth muscles ensues and, ultimately, the formation of vascularized connective tissue with smooth muscle cells, collagen, and endothelial cells (17, 29–31). These venous wall changes have been reported to occur hours to days after CVC insertion and are often progressive (31).

High blood flow rates through the central veins, often encountered in hemodialysis accesses, promote endothelial injury and, ultimately, stenosis (31). In addition, turbulent flow incites an inflammatory response and intimal hyperplasia culminating in venous wall remodeling. *De novo* CVS due to high blood flow rates were reported among six (10%) patients (out of 57 participants) in a study investigating the incidence of *de novo* CVS among HD patients. The average blood flow volume in four patients with measured access blood flow volume was 2347 mL/min (21). Similarly, two studies reviewed 69 and 103 patients for CVS and reported *de novo* cases among 14 and 64 patients, respectively (33, 34). Some of these incident cases may have had unreported central vein catheter placements or interventions, considering that CKD patients likely have high comorbid conditions that could predispose them to these interventions.

### Endoluminal obstruction

Thrombus associated with indwelling catheters can form within the vessel, obstructing blood flow or extraluminally. They arise from endothelial injury, flow turbulence, activation of the coagulation cascade, and fibrin sheath formation (35). Extraluminal thrombus, including right atrial or mural thrombus, can produce extrinsic catheter compression, resulting in insufficient blood flow and hemodialysis (35). Endoluminal obstruction may result from thrombosis, especially as a complication of the acute non-tunneled catheter relative to the chronic hemodialysis catheter. Most cases are subclinical and undetected, often diagnosed incidentally or in aggressive forms. As aforementioned, the presence of a foreign object in the vessel lumen precipitates thrombus formation, especially with prolonged indwelling. Without early treatment, the thrombus may become attached to the wall and organize to obstruct the lumen permanently (36).

### Extrinsic compression

Extrinsic compression is precipitated by tumor compression, post-surgical scarring, musculoskeletal compression, vascular compression, and fibrosis. The most frequent location of extrinsic mechanical compression of central veins is where the subclavian vein crosses between the clavicle and the first rib. In this instance, the thoracic outlet is relatively small; hence repetitive arm movement during exertion leads to progressive trauma to the endothelium and the wall of the subclavian vein inducing intraluminal thrombosis and causing stenosis (37).

## Clinical manifestations

**Figure 3:**
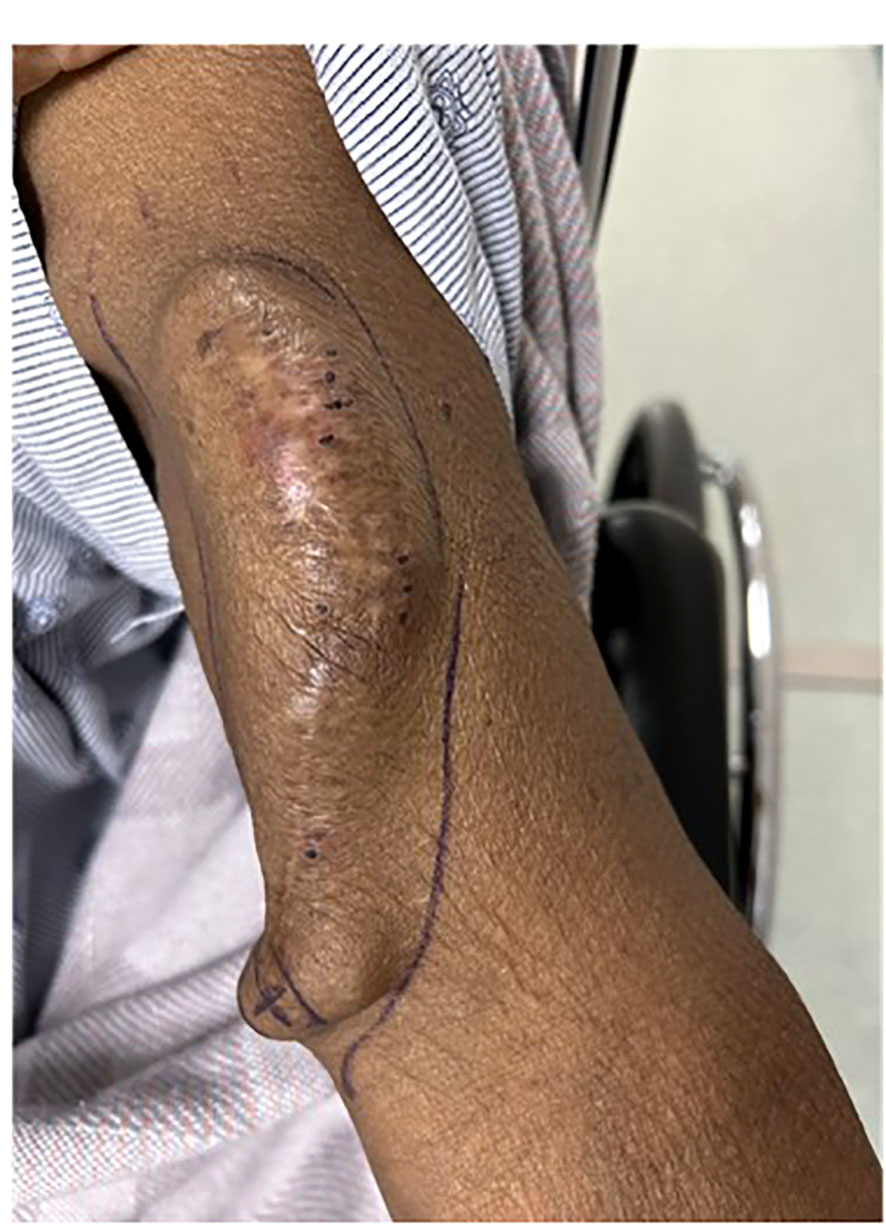
Left atrioventricular fistula HD access hypertension in a patient with central venous stenosis.

Central vein stenosis symptoms stem from venous hypertension behind the occlusion (**Figure 3**). Central venous stenosis can be asymptomatic. Signs are generally insidious in patients on HD but mostly become prominent in the presence of an ipsilateral arteriovenous graft or fistula draining into the affected central veins (38). CVS related to grafts and upper arm access are more likely to be symptomatic when compared to fistulas and forearm access (39). In functional HD access sites, asymptomatic CVS has been reported in up to 29% of cases (40). CVS may cause ipsilateral arm swelling, leading to severe venous dilatation, worsening upper extremity edema with pain and discomfort, skin ulceration, and recurrent infection if left untreated. The patient may also develop dilated and tortuous collateral veins over the ipsilateral arm, neck, and chest because the high venous blood flow and pressure via the fistula may overwhelm the collateral lymphatic and venous drainage (13, 41). Dialysis access sites become increasingly challenging to cannulate in clinically significant lesions due to vascular access thrombosis. Compromised blood flow, increased venous pressure during dialysis, excessive bleeding from the access site, and inadequate dialysis delivery due to access recirculation that eventually render the access inoperative are dreaded complications (38).

## Diagnosis

The diagnosis of CVS is suspected based on history and examination findings and confirmed by imaging. Important pointers in the history include risk factors such as a history of previous CVC placement and cardiac devices, complaints of arm swelling, pain or discomfort, skin ulceration, and access problems during dialysis (37, 42). Examination findings include ipsilateral arm edema and dilated collaterals in the neck or chest (37, 42). The clinical picture of SVC syndrome with facial edema can also be seen in bilateral SVC-related CVS (13). Multiple imaging techniques may be utilized to establish the diagnosis. Conventional venography is considered the gold standard for diagnosis but is invasive (40). Therefore, non-invasive imaging methods such as magnetic resonance (MR) angiography, computed tomography (CT) venography, and duplex ultrasound [DU] are often initially employed. KDOQI recommends central venous imaging before the creation of permanent vascular access in patients with ESRD suspected to have CVS or who have had prior CVC placement. It is required that venography be performed before treatment initiation (4, 13, 41).

Duplex ultrasound is cost-effective, non-invasive, can be used in patients with contrast allergy, and is easily reproducible. CVS is diagnosed on DU if the affected vessel fails to exhibit normal respiratory variation in vascular diameter and lacks polyphasic atrial waves (28). Limitations include the inability to fully visualize the proximal third of the subclavian or innominate vein; it also performs poorly in obese patients or those with significant muscle mass. DU is sensitive enough to identify clinically significant vein stenosis and in the presence of a pressure gradient of 3mmHg, a peak vein velocity ratio of >2.5 across the stenosis is the proposed best criterion (43). DU may also help select patients for intervention and monitor treatment success during follow-up (43). Stenosis severity is often determined by vascular diameter, with significant stenosis determined at >50 decreases in luminal diameter on ultrasonography. When utilized alone, the DU modality could present the risk of poorly estimating the lesion’s severity. Calcified vascular lesions, cross-section, and inappropriately high gain present technical limitations contributing to these estimation issues.1 Measures of peak velocity ratio, blood flow, and the residual diameter of 2 mm in grafts are additional criteria proposed to improve the diagnostic accuracy in determining the severity of central venous stenosis lesions (7, 44–46).

**Figure 4:**
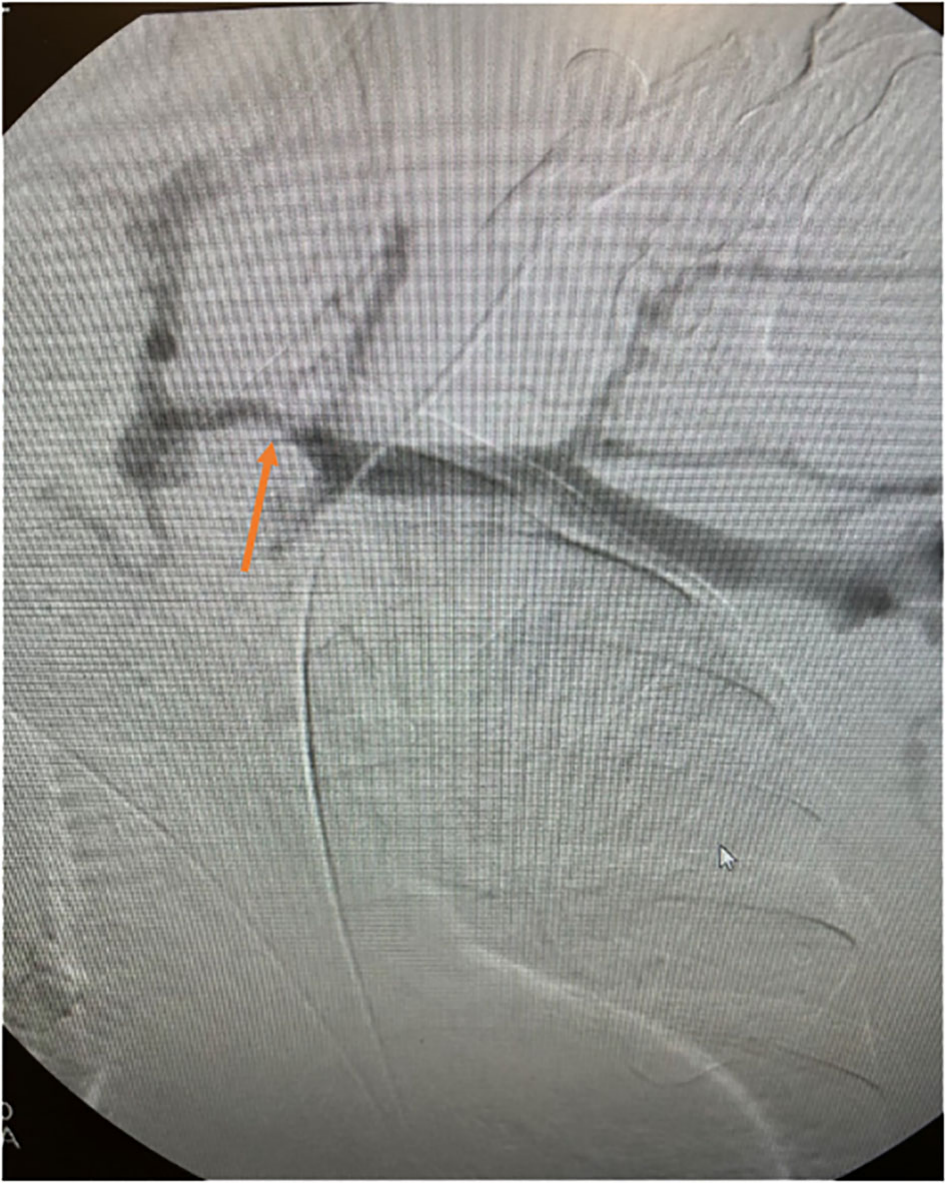
Angiographic representation of subclavian vein occlusion prior to revascularization.

Conventional catheter venography, either via digital subtraction or fluoroscopy, gives an exact outline of the central veins and reveals the presence of stenosis with localization of the lesion; a stenotic lesion greater than 50 percent is considered significant (**Figure 4**). The gold standard for CVS diagnosis is digital subtraction venography, which is more sensitive than DU (40, 47). MR venography is an alternative to conventional venography. It was initially discouraged in hemodialysis patients due to the risk of developing nephrogenic systemic fibrosis associated with administering gadolinium contrast (48). Recent studies utilizing ferumoxytol as an alternative report its safety in patients with renal impairment, making the use of MR venography feasible, with a sensitivity of 99% and specificity of 98% (49).

## Management strategies for symptomatic CVS associated with hemodialysis access

CVS management aims to relieve symptoms and morbidity and maintain vascular access longevity. Therapeutic intervention is only indicated in patients with clinically confirmed stenosis and associated symptoms (50). Stenotic lesions with a greater than 50 percent decrease in the luminal diameter are considered clinically significant (50). The presence of anatomic lesions without hemodynamic, functional, and clinical symptoms does not warrant prophylactic management and should only be observed (51). A retrospective study investigated the natural history of incidentally diagnosed high-grade CVS (>50%) among asymptomatic patients (52). This study treated 64 central venous stenosis lesions with percutaneous transluminal angioplasty, and 24 were left untreated. The authors observed an accelerated progression and *de novo* CVS lesions among the treatment population. In the study period, none of the patients in the untreated group exhibited clinical symptoms, *de novo* CVS, or progression of their CVS lesion, and none required intervention (52). The optimal management option depends on the nature and location of the lesion. There are no standardized trials comparing the techniques and outcomes of the various interventions.

## Endovascular interventions

KDOQI recommends PTA with or without stent placement as the preferred therapeutic approach for symptomatic CVS (53). Endovascular intervention for CVS has shown variable results but remains the recommended initial treatment in this patient population. Treatment options include percutaneous transluminal angioplasty with or without stents.

## Percutaneous transluminal angioplasty

**Figure 5:**
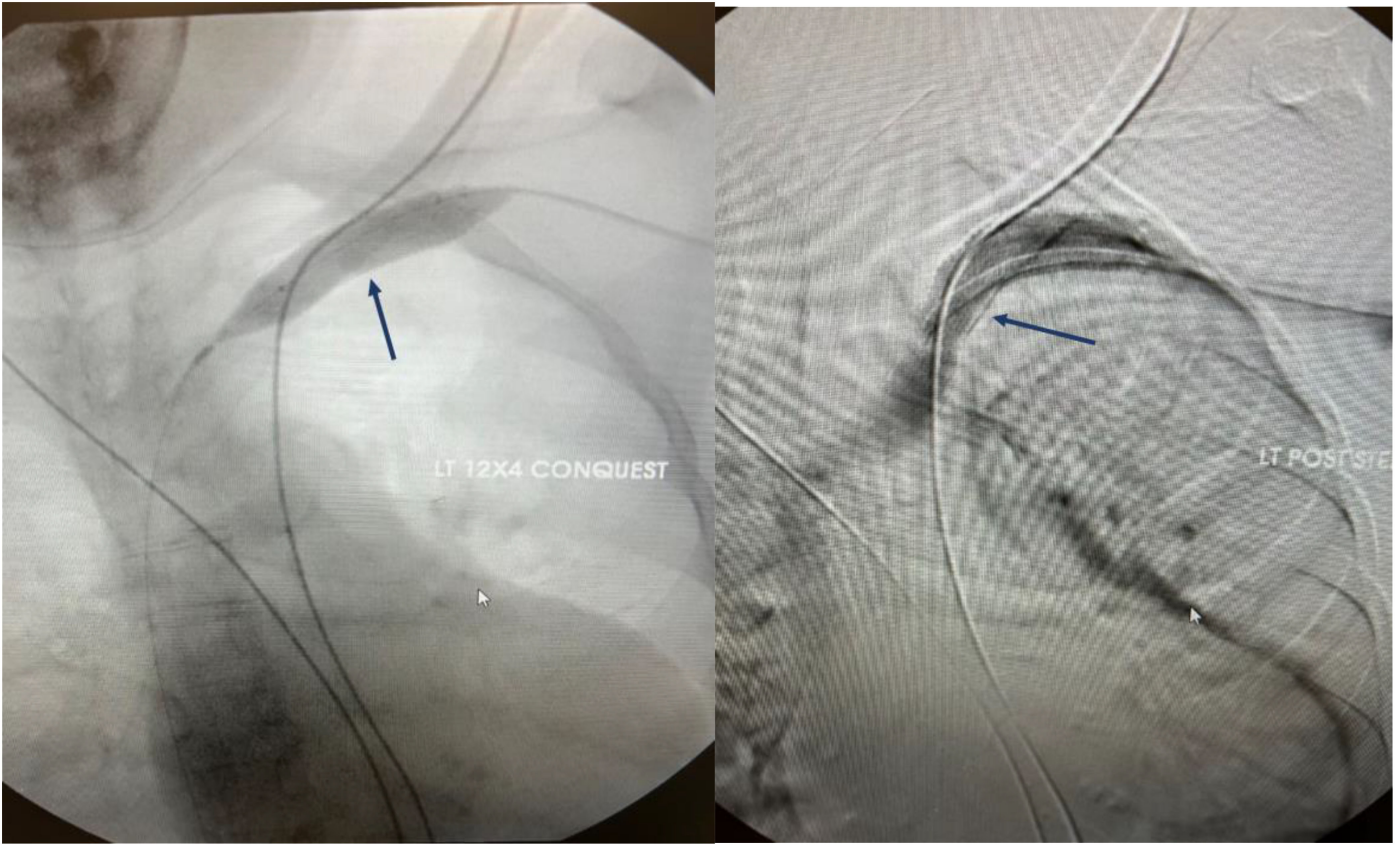
Angiographic representation of subclavian vein stenosis. The lesion was crossed with a sharp needle recanalization, then sequential a 10 x 8cm Conquest balloon was deployed followed by the 12x 4 cm Atlas balloon. A 13.5 x 10 cm Viabahn was ultimately deployed to maintain patency.

Angioplasty is the preferred treatment for symptomatic central vein stenosis (53) (**Figure 5**). The initial success rates, technical success, and complication rates are acceptable for PTA. No large, randomized trials have investigated PTA for the management of CVS. Based on the data from several studies, the reported technical success rate ranged from 70 to 90% (53–59). Unassisted patency rates following PTA ranged from 23% - 63% at 6 months, with cumulative patency rates ranging from 29%-100%. At 12 months, the unassisted patency rates were between 12% - 50% and 13-100% for cumulative patency (10, 54–59). However, these investigations employed different criteria to describe lesions, severity, and outcomes and were conducted in different demographics using diverse techniques resulting in substantial outcome heterogeneity (10, 54–59). Maintaining long-term patency and preventing occlusion necessitates repeated interventions due to a high recurrence rate. The high recurrence rate has partly been attributed to endothelial and vascular wall elasticity. Restenosis is not uncommon after endovascular interventions due to neointimal hyperplasia and often occurs at the same site (60). Angioplasty techniques require cracking and fissuring of the vessel intima, which can induce the recurrence of venous stenosis (61).

Davidson et al. presented the histologic characteristics of stenotic lesions seen in CVS (62). The authors employed catheter-based intravascular ultrasonography (IVUS) with contrast cine angiography. IVUS pictures were acquired during 38 successive percutaneous balloon angioplasties to manage hemodialysis fistula-related stenoses. Quantitative and qualitative evaluations were conducted on images of the vessels, including 11 central veins. Plaque dissection was observed in 16 (42%) lesions, and both vascular stretch and elastic recoil were seen in 19 (50%) patients. The combination of vascular stretch and dissection was reported in 7 (18%) cases, and elastic recoil and dissection occurred in nine (24%) patients. Central veins exhibited the most prevalence of elastic recoil, and this property accounts for high recurrence and inadequacy of initial PTA in maintaining patency (62). An immunohistochemistry study revealed a high proliferative index in the vessel wall (intima and media) among patients with re-stenotic lesions compared to those with primary stenosis (P<0.001). Diabetes was associated with an even greater risk of restenosis. Restenosis rates may be expedited by high blood flow rates and turbulence (60, 61). Following initial angioplasty, failure has been reported to exceed 30% residual stenosis (61). Patency rates have also been reported for angioplasty in managing cardiac pacemaker-induced CVS in HD patients. The primary patency rates were 18% and 9% at 6 and 12 months, respectively. At 6, 12, and 24 months, the secondary patency rates were 95%, 86%, and 73%, respectively. Secondary patency required an average of 2.1 procedures per year (63).

## Angioplasty with stents: bare metal stents and stent-grafts

The indications for stent placement in CVS remain the same for peripheral venous lesions. The guideline recommendation for stent deployment in managing CVS is for recurrent symptomatic lesions after PTA, especially within three months and those exhibiting elastic recoil (64, 65). (**Figure 5**) Stents resolve kinked stenotic lesions, prevent elastic rebound following balloon angioplasty, secure flow-limiting dissection, and maintain vein patency (53). It improves short- and long-term outcomes and HD access longevity. Self-expanding stents have shown superior success in managing elastic lesions than angioplasty alone (66). The inherent severity and nature of resistant lesions make comparing these populations challenging. Stent placement is not recommended in the device-related CVS due to a tendency for stent wire trapping in these cases. If stenting is indicated, the device should be removed and then replaced following placement (63).

Rajan et al. conducted a retrospective study of HD patients with autologous fistulas and synthetic grafts treated with angioplasties (83 angioplasty vs. 6 PTA with stents) for CVS to determine if primary patency rates differed across groups (67). Patients shared similar demographic characteristics with similar technical and clinical success. Previous ipsilateral central venous catheter placement was reported in about 76% of the patients. Primary patency rates ± standard errors at 3, 6, and 9 months were 88.5% ± 4.8, 59.4% ± 7.6, and 46% ± 7.9 rates in the fistula arm and 78.1% ± 7.3, 40.7% ± 9, and 16% ± 7.3, respectively. Overall, primary patency lasted longer for AV fistulas (p=0.014) and those with no prior history of CVC placement (p=0.001). Overall, endovascular interventions require repeat interventions to maintain patency and longevity of ipsilateral HD access sites.

In a retrospective study, Quaretti et al. compared the patency rates of various endovascular treatments for symptomatic central venous stenosis in 70 dialysis patients (68). A comparative analysis was conducted on three cohorts, including angioplasty alone (n=22), a bare metal stent (n=28), and stent graft (n=20). The stent graft demonstrated primary patency rates of 100%, 100%, 100%, and 84% at 3, 6, 12, and 24 months respectively, while angioplasty exhibited rates of 90%, 79%, 58%, and 43% (P = .014), and bare-metal stent showed rates of 84%, 80%, 75%, and 46% (P = .062). When the lesions’ sites were matched, the stent graft demonstrated a more favorable overall comparison (P = .020). There was no significant difference in angioplasty and bare-metal stent patencies (P = .141). The stent graft was associated with a lower risk of restenosis (hazard rate [HR] 0.20, confidence interval [CI] 0.06-0.7) and fewer reinterventions (P <.01). However, overall survival was influenced by age (HR 1.04, CI 1.001-1.08) and cardiovascular disease (HR 2.26, CI 1.06-4.84). No significant disparity was observed in the assisted primary patency. A prospective study compared the outcomes of CVS lesions treated with either PTA alone or with endovascular bare metal stent placement in patients undergoing hemodialysis. Eighty-seven patients were enrolled, 40 (46%) underwent PTA with stent placement, and 47 (54%) were treated with PTA alone. Primary patency rates with PTA were reported at 81%, 23%, and 12% at 60, 180, and 360 days, respectively, whereas the stent group achieved rates of 67%, 11%, and 11% at the same intervals (P =.4595). Secondary patency rates for PTA were 100% at 60, 180, and 360 days, respectively, whereas secondary patency rates for stents were 100%, 89%, and 78% (P =.5408) (59). There was no difference in the patency rates across the two interventions. High-pressure balloons are associated with about 60% and 30% primary patency rates at 6 and 12 months, better than previously reported (69). Primary patency rates at two years have been reported as low as 0% (55). Bakken et al. conducted a retrospective study comparing outcomes between HD patients with CVS who underwent high-pressure balloon angioplasty alone and PTA with stent group, primary, assisted primary patency, and ipsilateral HD survival were equal. The authors surmised that both techniques are safe and adoptable but were associated with high failure rates requiring repeat interventions.

Haage et al. reviewed the technical success, patency rates, and complications associated with stent placement in the primary management of central venous obstruction in hemodialysis patients (70). Fifty patients with symptoms of central venous obstruction underwent wall stent placement. There were no complications during stent deployment in any of the patients. One patient (2%) experienced an early re-thrombosis within one week. There were 73 cases of re-obstruction, of which 54 (74%) were treated percutaneously. Twenty-nine (26% of the cases) required additional stent placements. The primary patency rates were 92%, 84%, 56%, and 28% at 3, 6, and 12 months, respectively. Patency rates for the stents were 97% after 6 and 12 months, 89% after 24 months, and 81% after 36 and 48 months. Multiple interventions were required to maintain patency despite acceptable technical results from stent placement.

Similarly, a long-term study investigated the outcomes and effectiveness of stent-graft placement in managing CVS refractory to PTA in HD patients with functioning AV fistulas (71). Primary patency rates were 97%, 81%, 67%, and 45% at 3, 6, 12, and 24 months.

At 3, 6, 12, and 24 months, the primary assisted patency rates were 100%, 100%, 80%, and 75%, respectively. Patients who had not undergone PTA or bare metal stent placement had significantly shorter intervals to repeat intervention (P.018) than those who had previously undergone PTA or bare metal stent deployment. The primary patency interval was substantially shorter in patients with occlusive lesions (P.05) than in those with stenoses. Occluded veins were more likely to need additional stent grafts (P.02). Twelve patients needed additional stent grafts to preserve patency. In the case of covered stents, endothelialization is primarily facilitated by the graft material, which serves as an inert scaffold to prevent restenosis (71, 72). Despite this, the utilization of stent grafts should be individualized.

## Drug-eluting stents

To mitigate the high rates of post-intervention restenosis associated with PTA and bare metal stents, investigations are underway into specialized covered stents and drug-eluting stents (73). Paclitaxel-coated balloons (PCBs) have been evaluated for their effectiveness and safety in managing malfunctioning HD access in a few randomized studies and retrospective case reviews with promising results. Notably, many of these studies excluded patients with central venous stenosis, limiting the application of the results to this population (74–78). Kitrou et al. compared the clinically assessed intervention-free period of a paclitaxel-coated balloon with conventional balloon angioplasty to manage symptomatic CVS. A total of 40 patients were enrolled (with a mix of AVG and AVF HD access) into the balloon angioplasty group (N=20) vs. the PCB (N=20) group. Patients were followed for an average of 180 days. The median intervention-free period (IFP) was significantly better in the PCB group (PCB group: 179 days, vs. CBA group: 124.5 days, P= .026). Outcomes were similar in the two types of HD access, management of *de novo* or stenotic lesions, and those with prior CVC placement. Across the re-stenotic lesions in the PCB group, longitudinal comparison between treatments showed better outcomes in this group (median IFP in PCB group 177 vs. 91 days in CBA group; P= .01). Massmann et al. reported similar success with PCBA providing significant longevity from the need for revascularization compared to conventional balloon angioplasty (79, 80). Farber et al. compared outcomes between PTA and Dacron-covered nitinol stents in managing access-related venous occlusions (73). The authors noted a secondary patency rate of 60% at 3 and 6 months among the CVS (subclavian vein stenosis) cohort. This study is notably underpowered, and patients with peripheral venous lesions were included. A similar study reported cumulative patency rates of 67.7% and 55.4% at 6 and 12 months, respectively, for implantation of Dacron-covered stents (72).

## Access flow reduction with banding techniques

High-flow volumes across HD vascular accesses are linked to a high recurrence rate after initial interventional therapy. Patients may be asymptomatic and only experience severe symptoms when the overall cross-sectional area of draining collaterals is inadequate to manage the arterial flow. In this instance, some patients may require ligation of an otherwise well-functioning vascular access. Access inflow restriction techniques aimed at restoring flow balance can limit excess access blood flow and pressure to preserve the access function. Successful recurrence and symptom resolution prevention have been reported in patients with recalcitrant CVS lesions following angioplasty and stent placement by flow reduction via balloon-assisted banding of the inflow (21, 81). Patients with access to blood flow volume below 700-800 mL/minute may not have successful outcomes (81). Unsurprisingly, banded graft accesses’ reported primary patency rates are lower relative to AVF (82). Grafts with significantly occluded central venous outflow with no established collaterals are prone to recurrent thrombosis and imminent failure and should not be banded.

## Hemodialysis reliable outflow dialysis catheter

In patients with central vein occlusion but no conventional upper arm HD access alternatives, inserting a lower extremity graft or hybrid catheter-graft device is usually the next step. The HeRO graft is a composite graft that comprises a central venous silicon and nitinol outflow segment, which is inserted into the right atrium and connected to a polytetrafluoroethylene (PTFE) arteriovenous graft (83). An industry-funded randomized trial evaluated the safety and efficacy of the HeRO graft relative to the upper limb grafts. The study enrolled 72 patients, 20 in the graft and 52 in the HeRO cohorts. Quite notably, the investigators excluded patients with significant central venous stenosis. In addition, there was no significant difference in the 12-month primary and secondary patency rates in the HeRO and graft groups, 35% versus 31% and 68% versus 58%, respectively (84).

Particularly for CVS, Sur et al. conducted a retrospective investigation comparing the outcomes of HeRO graft and stent placement (85). The HeRO group included 29 patients, while 14 patients underwent stent placement. At follow-up of >500 days, primary patency among patients in the HeRO group was 16/28 (57%) and 4/14 (28%) in the stent cohort. The average number of interventions per patient year for the HeRo and stent groups were 1.4 and 2.3, respectively. There was no significant difference in the outcomes across both groups. The authors concluded that the HeRO graft is an alternative for HD patients with refractory CVS lesions who are poor surgical candidates. The HeRO graft may be an option in carefully selected patients in the right clinical setting, but many require at least two interventions per year to maintain patency and function.

## Surgical interventions

Open surgical techniques to treat central venous stenosis and occlusion are highly morbid, necessitating a median sternotomy to access deeply located central veins and the right atrium. These procedures often utilize autogenous veins or the polytetrafluoroethylene (PFTE) graft (86). Reported primary patency rates are high, approaching 80-85% at one year (87). Despite this high success rate, it has gained little enthusiasm due to its invasiveness and associated complications. The deep location of the central veins and the poor health status of these patients make it highly morbid (88, 89). It is regarded as a last resort in patients with failed endovascular interventions, young patients with minimal comorbidities, and refractory clinical symptoms (90).

Identifying the precise location of the lesion is critical to determining the best reconstruction technique. A central venogram is a necessary preoperative step to comprehensively map out the patient’s venous anatomy before open surgical intervention. The primary objective of surgical intervention is to establish venous outflow into the right atrium. Open surgical intervention can be achieved through central reconstruction, which involves connecting the central veins directly to the right atrium or via extra-anatomic graft bypass to the right atrium (89). The use of prosthetic grafts and spiraled great saphenous vein have been reported. In addition, open venous patch angioplasty has been documented as a viable approach for addressing stenotic central veins (91). The reported outcomes of these surgical options are positive. However, sternotomy-associated complications have led to their infrequent performance (88, 89). Doty et al. performed the first surgical intervention to manage HD-related CVS in a patient with superior vena cava syndrome by placing a spiral vein graft constructed from an autogenous vein. The patient had clinical relief and graft patency up to 6 months following the procedure (92).

Extra-anatomic bypass entails connecting the ipsilateral HD access to a peripheral vein draining into the right atrium. This avoids the morbidity and complications associated with a sternotomy. The saphenous, femoral, ipsilateral, and contralateral jugular veins have all been utilized as venous bypasses for access output (93, 94). However, in circumstances where the complete obstruction occurs, particularly at the osseous costoclavicular junction, the range of available endovascular interventions and open venous reconstructions are limited, necessitating the adoption of bypass grafting (95). Glass et al. presented the substernal tunneled subclavian to right atrial appendage bypass approach. This was performed in patients with occluded central veins, including the subclavian, innominate, and caval veins. These patients had occluded central veins with good fistula or symptomatic fistula malfunction, patent subclavian and axillary veins to the costoclavicular junction, and no alternative way to achieve HD access in the contralateral upper extremity. Intrathoracic access was gained by claviculectomy and “mini pericardiotomy” through the 3rd intercostal space exposing the right atrium. Three bypasses were performed with autogenous vein grafts (two femoral and one saphenous), while eight were performed with PTFE. The immediate postoperative complications were sepsis and acute pericardial effusion. The average follow-up duration was 16 months, and primary patency at 6 and 10 months were 67% and 33%, respectively. Notably, central bypass stenosis or occlusion rates appeared to be high at 36%, and postoperative infection rates were relatively high at 18%. Overall, there were significantly high failure and recurrence rates. Therefore, it could be pursued with extreme caution in very select patients with no other alternatives for HD access (86).

Obstructions affecting the innominate veins or the superior vena cava (SVC) are addressed by expanded polytetrafluoroethylene (ePTFE) grafts to create a bypass from either the axillary or subclavian veins to the jugular veins or right atrium (**Figure 3**). In addition, isolated subclavian vein occlusion can be treated with internal jugular vein turn-down or bypass graft. In IJV turn-down or transposition, the ipsilateral IJV is anastomosed to the distal subclavian or the axillary vein (94). The downside is that this procedure prevents IJVs from being used for hemodynamic monitoring, venous outflow for AV fistulas, or even temporary access in the future.

El-Sabrout et al. reported on right atrial bypass grafting outcomes for central venous occlusion among patients with previous bilateral temporary subclavian dialysis catheters (89). In their technique, a large diameter (10-16mm) externally reinforced PTFE graft bypassed the obstruction and was then anastomosed to the right atrial appendage. Clinical relief was recorded in 8 out of 9 patients following the procedure. However, grafts remained patent at an average of 15.4 (1.5-52) months. Although this study presented right atrial bypass grafting as a viable option in patients with central venous stenosis, the process of patient and technique selection was questioned (89).

Bhatia et al. compared the outcomes and patency rates between stent placement and surgical bypass graft. They reported similar symptom-free intervals in both groups at six and twelve months, with no significant difference in periprocedural complications and one-year mortality (87). This indicates that bypass graft should only be recommended for the minority of patients with no alternative access sites and lesions refractory to percutaneous angioplasty intervention (89).

Ayarragaray presented venous decompression as a novel surgical alternative for managing HD patients with central venous stenosis with PTFE graft malfunction (96). This procedure was performed on 3 HD patients. A 6 mm expanded and reinforced PTFE graft was connected to the brachiocephalic graft proximally, and the distal graft was connected to the femoral vein. There were no reported perioperative complications. Patients had clinical symptom improvement within the first 48-72 hours and had functional access to dialysis. At a follow-up of 16.3 months, one patient had clinically detectable AV graft dysfunction. The two other patients maintained graft patency till death. This procedure could be an option for patients with many vascular access points and significant central vein stenosis or blockage.

## Alternative renal replacement therapy strategies in CVS

In the event of bilateral recalcitrant thoracic CVS precluding installation of AV access in the upper limb, several options may be pursued for HD delivery in patients needing access. Complete or tight SVC stenosis often has high recurrence rates despite repeated and adequate endovascular interventions precluding future upper limb AVF or graft. Peritoneal dialysis should be considered when feasible. Upper thigh AVF or graft are also viable options. It is common for patients with high dialysis vintage to exhaust all viable definitive access options. In these instances, tunneled cuffed HD catheters and hemodialysis reliable outflow (HERO device) may be the alternatives (97). In the event of treatment failure, the only option is to occlude the AV access via balloon, manual, or surgical approaches. Occlusion leads to the resolution of symptoms associated with venous hypertension and precludes using the ipsilateral limb for access.

## Cardiac implantable electronic devices in hemodialysis

Evidently, cardiovascular complications abound among HD patients including arrhythmia and sudden cardiac death oftentimes requiring cardiac implantable electronic devices (CIED) such as pacemakers, loop recorders, and defibrillators (98). Patent central venous access is paramount for the creation of viable A-V HD access impacting survival. In instances where a patient has a CIED on one arm and no suitable veins for creating an arteriovenous fistula on the other arm, the process of creating permanent vascular access can become complicated. This is particularly challenging when hemodialysis is the only available option for renal replacement therapy. The risks associated with creating arteriovenous access on the side with the CIED include venous hypertension resulting from central vein stenosis related to the leads and the potential for systemic infection, including lead-associated endocarditis (99). In some instances, placement of CIED can be precluded by pre-existing stenosis stemming from HD vascular access complications. Consequently, the various clinical scenarios of CIED placement in HD patients require individualized management demanding a multidisciplinary collaboration between the nephrologist and cardiologist. Innovations in the CIED realm have provided alternative device options to standard pre-pectoral in this patient population (99). Alternative routes of placement such as endovascularly placed leadless pacemakers, subcutaneous implantable cardioverter defibrillators, and endocardial left ventricular pacing options are available to patients with CV stenosis or those in whom vascular preservation for future AV hemodialysis access is anticipated (99). Likewise, in HD patients with bilateral subclavian vein occlusion/superior vena syndrome, the femoral or iliac pacing system could be considered.

## Conclusion

Hemodialysis patients with a history of indwelling central venous catheter or intravascular device placement are at risk for central venous stenosis. Patients are often asymptomatic, but appropriate diagnostic investigations should be pursued in those with clinical indications. A complex vascular dilemma may result from coexisting cardiac implantable devices and arteriovenous fistulae, grafts, and tunneled catheters in hemodialysis patients. Perhaps the use of leadless pacemakers, epicardial leads, and subcutaneous defibrillators will lead to a reduction in the incidence of central venous stenosis in patients with ESRD. Treatment is reserved for clinically significant lesions, and percutaneous angioplasty is the preferred initial form of therapy. Recurrence and recalcitrant lesions abound, requiring multiple interventions to maintain patency and functioning HD access. In these cases, surgical bypass of the obstruction site may be required. Further prospective, randomized controlled studies with extended follow-up of currently available therapeutic options are needed to develop superior management protocols.

## Author contributions

IS: Writing – original draft, Writing – review & editing. GE: Conceptualization, Writing – original draft, Writing – review & editing. AL: Writing – review & editing. GS: Writing – review & editing. IB-R: Writing – review & editing. LG: Writing – review & editing. JS: Writing – review & editing. DK: Conceptualization, Writing – original draft, Writing – review & editing.
